# Comparison of Two Protocols for the Assessment of Maximal Respiratory Pressures: Spanish Society of Pulmonology and Thoracic Surgery Versus American Thoracic Society/European Respiratory Society

**DOI:** 10.7759/cureus.19129

**Published:** 2021-10-29

**Authors:** Ana Lista-Paz, Sergio Sancho Marín, Sonia Souto Camba, Cristina Jácome, Luz González Doniz

**Affiliations:** 1 Faculty of Physiotherapy, The University of A Coruña, A Coruña, ESP; 2 Faculty of Medicine, University of Porto (FMUP), Center for Health Technology and Services Research (CINTESIS), Porto, PRT

**Keywords:** physical therapy modalities, breathing exercises, respiratory function tests, respiratory muscles, maximal respiratory pressures

## Abstract

Background

The measurement of maximal respiratory pressures (MRPs) is commonly used to assess respiratory muscle strength. However, in Spain, there is no consensus on which is the most adequate measurement protocol, as theSpanish Society of Pneumology and Thoracic Surgery (SEPAR) protocol differs from the one endorsed by the American Thoracic Society/European Respiratory Society(ATS/ERS). This study compared the absolute and predictive values of maximal expiratory and inspiratory pressures (MEP and MIP) in healthy adults obtained with the two protocols.

Methods

A cross-sectional study with a sample of healthy adults was conducted. Lung function and MRPs were assessed. MEP and MIP were measured using a digital manometer according to the SEPAR and ATS/ERS. Protocols were applied in random order by the same trained physiotherapist. The comfort experienced with each protocol was assessed through a short questionnaire. Paired t-tests were used to compare the results from both protocols.

Results

A total of 31 subjects (mean age 35.7±12.4 years; 14 females; FEV_1_=108.3±10.5%; FVC=103.7±10%) were included. There was a significant difference between MRPs favouring the SEPAR protocol, with the mean difference being 34.9±28.1 cmH_2_O (p˂0.001) for MEP and 8±11.6 cmH_2_O (p=0.001) for MIP. ATS/ERS protocol was, however, considered more comfortable than SEPAR (p<0.005).

Conclusions

This study shows that, in healthy adults, higher MRPs are obtained using the SEPAR protocol. Yet, the ATS/ERS protocol is experienced as more comfortable. Future studies are needed to analyse the application of both protocols in other populations and their associated comfort.

## Introduction

Maximal static respiratory pressures are non-invasive measures to assess respiratory muscle strength with high value in the clinical management of patients with respiratory diseases [1]. Pulmonary rehabilitation is an evidence-based intervention for patients with chronic obstructive pulmonary disease (COPD) and one of its components is the assessment and training of respiratory muscle strength. In patients with neuromuscular diseases, it is also important to determine the strength of respiratory muscles to analyse the effectiveness of cough [2,3]. There are other clinical fields, in which the relevance of maximal respiratory pressures (MRPs) is growing, for example, in people who have suffered a stroke, as they present significantly lower maximal expiratory and inspiratory pressures (MEP and MIP) and its specific training has shown to be effective [4,5]. Assessment of MIP is also important to determine the timing for weaning patients off mechanical ventilation [6]. The relevance of respiratory muscle strength assessment is, however, not restricted to specific conditions. There is also evidence about the benefits of respiratory muscle assessment and training in athletes and healthy subjects [7]. Despite the available evidence demonstrating that evaluation of MRP is essential to diagnose respiratory muscle dysfunction and to prescribe the intensity of respiratory muscle training, there is no consensus on which evaluation protocol is the most adequate [8,9].

We find in the literature several differences in the procedures used to evaluate MRP that can affect the results: the type of mouthpiece and manometer; presence and size of a small leak; the number of repetitions performed, taking into account the learning effects and the fatigue; manoeuvre duration; percentage of variability between repetitions; the pressure measured (peak or plateau), among others [9-15]. To overcome the limitations associated with these differences, standardized assessment protocols were developed: the national Spanish Society of Pneumology and Thoracic Surgery protocol (SEPAR) and the joint international statement from the American Thoracic Society/European Respiratory Society (ATS/ERS) [1,16]. These protocols have relevant differences that may affect the results obtained and their clinical interpretation. This can lead to errors in identifying patients with respiratory muscle weakness and in providing adequate intervention, with the required load during respiratory muscle training. Health professionals feel, therefore, unsure about which protocol should be used in their clinical practice. On the other hand, patients’ experience with each protocol is rarely addressed.

The main aim of this study was thus to compare the absolute and predictive values of MEP and MIP obtained following the SEPAR protocol and the ATS/ERS protocol in a sample of healthy Spanish adults. Our secondary aims were to explore differences in identifying respiratory muscle weakness and differences in the participants’ experienced comfort.

## Materials and methods

Study design

A cross-sectional study was conducted in a sample of at least 30 healthy Spanish adults. The study is reported according to Strengthening the Reporting of Observational Studies in Epidemiology (STROBE) guidelines [17]. This project was approved by the ethics committee of the University of A Coruña. All subjects signed a consent form before any data collection.

Participants and setting

Subjects were invited to participate in the study via A Coruña University generic mailing list, from March to April 2018. Subjects were included if they were aged between 20 and 80 years old, Caucasians, and demonstrated a willingness to participate [18]. Exclusion criteria included current or former (for less than a year) smokers, people who had a diagnosis of a pulmonary, severe cardiovascular, cerebrovascular, and/or neuromuscular disease; had undergone thoracic or abdominal surgery in the previous three months; presented chest infections in the previous two months; severe thoracic wall deformities; obesity over 20% of overweight according to body mass index (BMI); pregnant women; professional athletes; people who showed altered forced spirometry according to the international guidelines and those who did not understand the instructions for the tests [19]. People who presented contraindications for any of the tests included in this protocol were also excluded [1,16,19]. Additionally, we excluded specialists in Respiratory Therapy at the University, since they are familiarized with both protocols for the measurements of MRP and this could introduce a selection bias.

Ninety-nine subjects replied to the invitation; then as we aimed a sample of at least 30 people with distinct characteristics (sex and age), and due to time and equipment restrictions, we randomly selected 32 adults using Excel software. If some of the randomly selected participants present any exclusion criterion, this subject was randomly replaced by another subject in the list.

Procedure

All assessments were carried out by a trained physiotherapist at the Faculty of Physiotherapy, University of A Coruña. During a single visit, all participants underwent a standardized interview regarding their demographic characteristics, history of smoking, and possible clinical antecedents. Height, weight, and BMI were also recorded. To characterize physical activity, in order to check if the physical activity behaviour of our small sample would be heterogeneous as it is the Spanish population, the validated Spanish version of the International Physical Activity Questionnaire (IPAQ) short version was used [20].

Then, in order to identify possible alterations in lung function, forced spirometry was performed in accordance with the international guidelines using a Datospir®120C spirometer [19]. The reference values for the Spanish population were calculated using the predictive equations proposed by Roca et al. [21]. At this point, if an altered lung function was detected, considered as FEV_1_/FVC<0.7 and FVC <80%, subjects were excluded [19].

MEP and MIP were measured using a digital manometer (model 511-8D0-MU1, Sibel Group, Spain) with an operating interval of ±300 cmH_2_O and a precision of 3% connected to a spirometer Datospir®120C. Equipment calibration was performed before each session of work. All measurements were taken in a sitting position with a rubber diver nozzle (scuba mouthpiece, model 05602, Sibel Group, Spain). MEP was measured before MIP in both protocols since it is an easier manoeuvre. MEP was performed from total lung capacity and MIP from residual volume [1,16]. The two protocols (SEPAR and ATS/ERS) were performed by all subjects in random order to avoid the learning effect and fatigue [12]. A ten-minute rest occurred between protocols. All participants were vigorously encouraged to make maximal efforts during the tests. Differences between SEPAR and ATS/ERS protocol are detailed below.

Maximal Respiratory Pressures Assessed Following SEPAR Protocol

Nose clips were used during the SEPAR protocol. We asked participants to sustain pressures for three to five seconds. During the MEP, subjects were asked to hold their cheeks rigid with their hands to avoid leaks and to minimize buccinators' muscle contribution during the manoeuvres. We performed a maximum of 10 repetitions to measure MEP and MIP, with a minimum of six acceptable manoeuvres (defined as manoeuvres without air leaks and with the graph showing a trend to a plateau), three of them with variability <5% (repeatability criteria). We chose the highest reading of the three reproducible manoeuvres [16]. The participants had to rest for one minute between each repetition of MEP and five minutes before starting the MIP evaluation [16].

Maximal Respiratory Pressures Assessed Following ATS/ERS Protocol

Nose clips were not used during the ATS/ERS protocol, and participants did not hold their cheeks with their hands during MEP. We asked volunteers to sustain the maximal effort for 1.5 seconds. We looked for three acceptable manoeuvres, with variability below 20%, and we chose the higher one [1].

The predictive values for MEP and MIP were calculated according to the equations proposed for the Spanish population by Morales et al., as well as by Wilson et al., for Caucasian-European [18,22].

Comfort experienced during manoeuvres

The comfort experienced during manoeuvres was assessed after each protocol through a self-reported four-item questionnaire designed by the researchers for that purpose (Appendix 1). Each item was rated on a 5-point Likert scale from 1 (no comfort at all) to 5 (very comfortable). The internal consistency of the questionnaire was verified [23]. A Cronbach’s alpha of 0.86 was found, which indicates a high level of internal consistency for our scale.

Data analysis

Demographic continuous variables are presented as mean and standard deviation (SD), while categorical values are shown as absolute values and percentages. MEP and MIP absolute values obtained with both protocols were compared with the reference values available for the Spanish and European population and respiratory muscle weakness was interpreted as MIP and/or MEP below 65% of reference value [18,22,24]. After testing the normal distribution of data using the Shapiro-Wilk test, paired t-tests were used to explore differences in MEP and MIP values between the two protocols [25]. Fisher’s exact test was used to compare the frequency of respiratory muscle weakness detected by each protocol. To compare the results obtained with the comfort questionnaire, Wilcoxon tests were used. A significant level was set at P<0.05. All statistical analyses were performed using Statistical Package for Social Sciences (SPSS) software version 24.0 for Windows (IBM S.A., Madrid, Spain).

## Results

Thirty-two evaluations were scheduled, but one subject did not attend the appointment. Seventeen males and 14 females were assessed (35.7±12.4 years). There were only three ex-smokers in the sample, and all the subjects had a normal lung function (FEV_1_/FVC=0.8±0.1; FVC=103.7±10%). Subjects’ characteristics are given in Table 1.

**Table 1:** Characteristics of the subjects (n=31) Values are expressed as mean ± standard deviation unless otherwise indicated. Lung function parameters are expressed as an absolute value and as a percentage of the reference values calculated using the predictive equations proposed by Roca et al. [23]. BMI: body mass index; cm: centimetre; FEF25-75%: forced expiratory flow at 25-75% of the FVC; FVC: forced vital capacity; FEV1: forced expiratory volume in one second; IPAQ: International Physical Activity Questionnaire; kg: kilograms; l: litres; l/s: litres per second; PEF: peak expiratory flow.

Characteristics	
Males/females, n (%)	17(55)/14(45)
Age (years)	35.7±12.4
Anthropometry	
Weight (kg)	73.6±14.4
Height (cm)	169.8±7.9
BMI (kg/m^2^)	25.4±3.6
IPAQ-short form	
Total METs (min/week)	2985.3±2143
Total time spent sitting (h/week)	7.1±2.8
High physical activity, n (%)	16(51.6)
Moderate physical activity, n (%)	13(41.9)
Low physical activity, n (%)	2(6.5)
Lung function	
FEV_1_ (l)	3.9±0.8
FEV_1_ (%)	108.3±10.5
FVC (l)	4.8±1
FVC (%)	103.7±10
FEV_1_/FVC (%)	82.3±6.3
PEF (l/s)	8.7±1.7
PEF (%)	104.4±12.7
FEF_25%-75% _(l/s)	4.1±1.3
FEF_25%-75% _(%)	110.2±27.3

Table 2 shows the results of MEP and MIP for both protocols as a mean of absolute value and as a percentage of the predictive value, based on the equations of Morales et al. and Wilson et al. [18,22]. A significant difference both in MEP (mean difference=34.9±28.1 cmH_2_O; 95%CI=24.6-45.2; p<0.001) and MIP (mean difference=8±11.6 cmH_2_O; 95%CI=3.8-12.3; p=0.001) was found, with higher values for the SEPAR protocol. According to the equation of Morales et al., the mean of MEP was 71.9±18.8% of reference value following the SEPAR protocol and 52.4±11.5% following the ATS/ERS protocol [18]. For MIP values, we obtained 71.5±17.2% of reference value with the SEPAR protocol and 66.1±17.1% with the ATS/ERS protocol. Using the aforementioned equation, with SEPAR protocol, 42% of participants were identified with expiratory and 42% with inspiratory weakness; while with ATS/ERS higher frequencies were found, 61% (p=0.003) and 87% (p=0.120), respectively [18]. Significant differences were also found between SEPAR and ATS/ERS protocols for both, MEP and MIP, when comparing the percentages of predictive values calculated with Wilson’s equations [22]. With these equations, with SEPAR protocol no participant was identified with expiratory weakness, while with ATS/ERS protocol 26% of subjects showed expiratory weakness [22]. Regarding MIP, only 3% showed an inspiratory weakness with SEPAR protocol and 16% with ATS/ERS protocol (p=0.161). Notably, 19 subjects spontaneously narrated a subjective feeling of air leaks during measurements with the ATS/ERS protocol, but none reported the same with the SEPAR protocol.

**Table 2:** Comparison between maximal respiratory pressures obtained with SEPAR and ATS/ERS protocols using paired t-tests (n=31) *Statistical significance. Reference values of maximal respiratory pressures were calculated using predictive equations proposed by: ^a^Morales et al.; ^b^Wilson et al. [18,22] ATS/ERS: American Thoracic Society/European Respiratory Society; cmH_2_O: centimetres of water; 95%CI: confidence interval 95%; MEP: maximal expiratory pressure; MIP: maximal inspiratory pressure; SEPAR: Spanish Society of Pneumology and Thoracic Surgery

Variable	SEPAR (n=31)	ATS/ERS (n=31)	Mean difference±SD	95%CI	p
	Mean±SD	Mean±SD			
MEP (cmH_2_O)	128.3±43.7	93.4±25.3	34.9±28.1	24.6–45.2	<0.001>
MEP refence values (cmH_2_O) Morales’ equations/Wilson’s equations	179.3±41.3/122.4±28		-	-	-
MEP (%)^a^	71.9±18.8	52.4±11.5	19.5±13.4	14.6–24.4	<0.001>
MEP (%)^b^	104.8±26.6	77.2±16.7	27.6±19.3	20.5–34.7	<0.001>
MIP (cmH_2_O)	90.7±24.1	82.7±23.7	8±11.6	3.8–12.3	0.001*
MIP refence values (cmH_2_O) Morales’ equations/Wilson’s equations	125±23.5/90±18.4		-	-	-
MIP (%)^a^	71.5±17.2	66.1±17.3	5.4±9.7	1.8–9	0.004*
MIP (%)^b^	101.5±21.8	92.6±22.6	8.9±13.1	4.1–13.7	0.001

**Figure 1:**
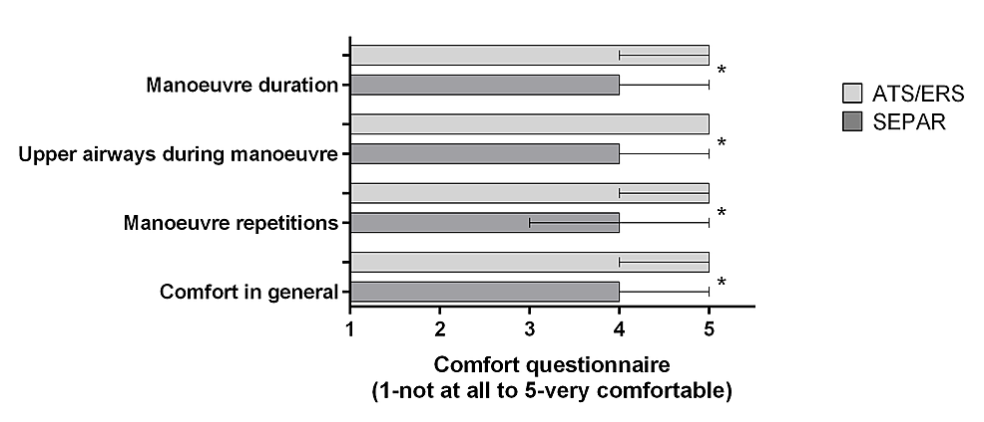
Results of the four-item comfort questionnaire Data are presented as median and interquartile ranges. Significant differences are identified with *(P<0.05).

Regarding the comfort questionnaire, as can be seen in Figure 1, ATS/ERS protocol was experienced as more comfortable compared to the SEPAR protocol in relation to the four-items assessed (medians 5 vs. 4).

## Discussion

The results of this study demonstrated statistically significant differences in both MEP and MIP absolute and predictive values assessed following the international protocol proposed by ATS/ERS and the Spanish protocol from SEPAR, showing higher results with the latter. In light of these findings, the SEPAR protocol was demonstrated to be more suitable for the measurement of MRP in the Spanish population. However, a higher comfort seems to be associated with ATS/ERS protocol. These findings support a deeper reflection on the measurement protocols for MRP is indeed needed.

The mean differences between both protocols are statistically significant for both MEP and MIP, but it is substantially higher for MEP and with a wide 95%CI, from 24.6 cmH_2_O to 45.2 cmH_2_O. The clinical implications of these differences are still unknown. First, because there is no consensus in the literature about how to express the results, given that some authors report absolute values (commonly in cmH_2_O), others the percentage in relation to reference values and, more recently, some authors highlight the importance of using the lower limit of normal values. But there is also no consensus on which cut-points should be used to identify respiratory muscle dysfunction [8,9,26]. International standards establish that absolute values higher than 80 cmH_2_O exclude respiratory muscle weakness [1]. More recently, guidelines for the assessment of muscular dysfunction in patients with COPD state that respiratory muscle weakness exists when MIP and/or MEP is below 65% of reference value [24]. According to this most recent criterion, we would exclude expiratory muscular weakness in some of the participants when they were assessed according to the SEPAR protocol, since on average 72% of reference value was achieved, and 42% showed a MEP˂65% reference value calculated with the equations by Morales et al. [18]. Yet, possibly, we would classify some of these healthy subjects as having expiratory muscle weakness when the ATS/ERS protocol was performed, given that average MEP was 52% of the reference value, and 87% had an MEP value ˂65% or reference value [18]. At that point, is also worthy to mention that it has been demonstrated that the Spanish reference equations proposed by Morales et al. significantly overestimated the real value of healthy Spanish adults, especially for MEP [18,27]. That is why Wilson’s equations were also calculated, in order to confirm the healthy condition of our sample [22]. Second, achieving an accurate measurement of MRP is highly important in order to prescript an efficient respiratory muscle training program, given that the external load imposed on these muscles must be based on basal MEP and MIP. Third, the scientific community needs evidence of which is the most suitable protocol for the assessment of MRP in order to apply it when new reference equations are created [24]. These examples of the clinical and scientific implications of using two distinct protocols, clearly demonstrate the need to follow a single and standardized protocol. This can be achieved, for example, by working with international partners and developing a new protocol merging the most adequate procedures of the two protocols.

Previous evidence shows that differences in procedures for assessing the MRP can determine values obtained [8,9]. However, as far as we know, this is the first study conducted to compare the international protocol-ATS/ERS with a national protocol. One of the main differences between both protocols that could affect MEP and MIP values is the repeatability criteria (a minimum of six acceptable manoeuvres, three of them with variability <5% with SEPAR vs. a minimum of three acceptable manoeuvres with variability <20% with ATS/ERS) [1,16]. When we had to register a minimum of six acceptable manoeuvres with a small range of variability between three of them (SEPAR protocol), participants had more opportunities to learn the technique since we had to repeat more times to achieve that criteria and this could be one of the principal reasons for the higher results with the SEPAR protocol, given that the learning effects have been previously shown [12,13]. Following the SEPAR protocol, we performed the maximum allowed repetitions (10 trials) with most participants in order to obtain the higher and reproducible value. However, following the ATS/ERS protocol three trials were enough for all subjects to obtain acceptable and reproducible manoeuvres within the acceptable range of 20% of the variability.

Apart from repeatability criteria, we believe that there were another two important factors that could explain differences in MEP. In the ATS/ERS protocol, the nose clips are not required and participants do not hold their cheeks rigid with their hands, conditions presented in the SEPAR protocol to avoid leaks [1,16]. Indeed, the authors noticed how difficult it was for subjects to avoid air leaks around their lips during the ATS/ERS protocol. In fact, 19 volunteers spontaneously reported a subjective feeling of air leaks during measurements with the ATS/ERS protocol, but none reported the same with the SEPAR protocol.

Differences in MIP values were also detected, but with a lower extent in comparison with MEP. MIP was 72% of reference value with the SEPAR protocol and 66% of reference value with ATS/ERS (which is very near the 65% to consider inspiratory muscle weakness) when Morales’ equations were used. However, our participants showed normal percentages of MIP when Wilson’s equations were used [18,22]. It is also worthy to mention that the ATS/ERS protocol was experienced as comfortably as the SEPAR protocol by the participants in this study. This aspect should be also considered in different populations that normally experience more fatigue than healthy people, such as, patients with respiratory diseases, neuromuscular diseases, or after a stroke.

This study has some limitations that should be acknowledged. Despite having found statistically significant differences in reference values for MEP and MIP comparing SEPAR and ATS/ERS protocols, with clinical relevance in the case of MEP, we do not know if these results can be generalized to the healthy population or even if it applies the same way to other populations. It would be interesting to replicate this study in patients with COPD or patients with neurological diseases since these patients are the ones who more often need respiratory muscle training [5,28,29]. Also, as we used two different established protocols, we cannot distinguish which differences between them affect more the results of MEP and MIP (manoeuvre duration, number of repetitions, repeatability criteria, use of nose-clips, and hands placed over the checks). Hence, we could consider this work as the first step for the analysis of the best protocol to achieve the most reliable results of MRP. Second, recently ERS has published an updated statement on respiratory muscle testing where the variability between manoeuvres has decreased from 20% to 10% [30]. This shows that the international scientific community is reflecting on the necessity to improve the measurement protocol for MEP and MIP. Taking these findings into account, we should review internationally the available protocols for assessing MRP. From our point of view, a standardization effort between ATS/ERS and SEPAR protocols must be explored in future investigations. Finally, the reliability of protocols was not assessed in our study, and this should be explored in a future study with a large sample.

## Conclusions

MEP and MIP values are significantly higher when we use the SEPAR protocol than when we use the ATS/ERS protocol in healthy adults, especially for MEP. However, the ATS/ERS protocol was experienced as more comfortable. According to our results, it would be advisable to review the international protocol for measuring MRP in healthy people for the determination of new reference equations, but also in other populations.

## Appendices

Comfort questionnaire for maximal respiratory pressures assessment

This short questionnaire aims to assess the degree of comfort experienced during the assessment of maximal respiratory pressures. Please rate each question from 1 (no comfort at all) to 5 (very comfortable).

1. Grade how comfortable was the duration of the maneuver? (by duration we mean the required time to maintain the inspiration or expiration)?

No comfort at all  1 o        2 o        3 o        4 o        5 o  Very comfortable

2. Grade how comfortable you felt in relation to your upper airways (nose)?

No comfort at all  1 o        2 o        3 o        4 o        5 o  Very comfortable

3. Grade how comfortable you felt in performing the required repetitions for each maneuver?

No comfort at all  1 o        2 o        3 o        4 o        5 o  Very comfortable

4. In general, grade how comfortable you felt during these maneuvers?

No comfort at all  1 o        2 o        3 o        4 o        5 o  Very comfortable

## References

[1] (2002). ATS/ERS statement on respiratory muscle testing. Am J Respir Crit Care Med.

[2] (2020). Global strategy for the diagnosis, management, and prevention of chronic obstructive pulmonary disease: Global initiative for chronic obstructive lung disease. https://goldcopd.org/wp-content/uploads/2019/11/GOLD-2020-REPORT-ver1.0wms.pdf.

[3] Sferrazza Papa GF, Pellegrino GM, Shaikh H, Lax A, Lorini L, Corbo M (2018). Respiratory muscle testing in amyotrophic lateral sclerosis: a practical approach. Minerva Med.

[4] Lista Paz A, González Doniz L, Ortigueira García S, Saleta Canosa JL, Moreno Couto C (2016). Respiratory muscle strength in chronic stroke survivors and its relation with the 6-minute walk test. Arch Phys Med Rehabil.

[5] Wu F, Liu Y, Ye G, Zhang Y (2020). Respiratory muscle training improves strength and decreases the risk of respiratory complications in stroke survivors: a systematic review and meta-analysis. Arch Phys Med Rehabil.

[6] Elkins M, Dentice R (2015). Inspiratory muscle training facilitates weaning from mechanical ventilation among patients in the intensive care unit: a systematic review. J Physiother.

[7] Karsten M, Ribeiro GS, Esquivel MS, Matte DL (2018). The effects of inspiratory muscle training with linear workload devices on the sports performance and cardiopulmonary function of athletes: a systematic review and meta-analysis. Phys Ther Sport.

[8] Evans J, Whitelaw W (2009). The assessment of maximal respiratory mouth pressures in adults. Respir Care.

[9] Sclauser Pessoa IM, Franco Parreira V, Fregonezi GA, Sheel AW, Chung F, Reid WD (2014). Reference values for maximal inspiratory pressure: a systematic review. Can Respir J.

[10] Montemezzo D, Vieira DS, Tierra-Criollo CJ, Britto RR, Velloso M, Parreira VF (2012). Influence of 4 interfaces in the assessment of maximal respiratory pressures. Respir Care.

[11] Mayos M, Giner J, Casan P, Sanchis J (1991). Measurement of maximal static respiratory pressures at the mouth with different air leaks. Chest.

[12] Terzi N, Corne F, Mouadil A, Lofaso F, Normand H (2010). Mouth and nasal inspiratory pressure: learning effect and reproducibility in healthy adults. Respiration.

[13] Volianitis S, McConnell AK, Jones DA (2001). Assessment of maximum inspiratory pressure. Prior submaximal respiratory muscle activity ('warm-up') enhances maximum inspiratory activity and attenuates the learning effect of repeated measurement. Respiration.

[14] Man WD, Kyroussis D, Fleming TA (2003). Cough gastric pressure and maximum expiratory mouth pressure in humans. Am J Respir Crit Care Med.

[15] Windisch W, Hennings E, Sorichter S, Hamm H, Criée CP (2004). Peak or plateau maximal inspiratory mouth pressure: which is best?. Eur Respir J.

[16] Calaf N (2011). Procedimientos de evaluación de la función pulmonar II [Internet]. Manual SEPAR de Procedimientos.

[17] Vandenbroucke JP, von Elm E, Altman DG (2014). Strengthening the reporting of observational studies in epidemiology (STROBE): explanation and elaboration. Int J Surg.

[18] Morales P, Sanchis J, Cordero P, Díez J (1997). Maximum static respiratory pressures in adults. Reference values for a Caucasian Mediterranean population [Spanish]. Arch Bronconeumol.

[19] Graham BL, Steenbruggen I, Miller MR (2019). Standardization of spirometry 2019 update. An official American Thoracic Society and European Respiratory Society technical statement. Am J Respir Crit Care Med.

[20] Roman-Viñas B, Serra-Majem L, Hagströmer M, Ribas-Barba L, Sjöström M, Segura-Cardona R (2010). International physical activity questionnaire: reliability and validity in a Spanish population. Eur J Sport Sci.

[21] Roca J, Sanchis J, Agusti Vidal A (1986). Spirometric reference values from a Mediterranean population. Bull Eur Physiopathol Respir.

[22] Wilson SH, Cooke NT, Edwards RH, Spiro SG (1984). Predicted normal values for maximal respiratory pressures in caucasian adults and children. Thorax.

[23] Mokkink L, Prinsen C, Patrick D, Alonso J, Bouter L, Vet H, Terwee C (2018). COSMIN guideline for systematic reviews of patient-reported outcome measures. Qual Life Res.

[24] Barreiro E, Bustamante V, Cejudo P (2015). Guidelines for the evaluation and treatment of muscle dysfunction in patients with chronic obstructive pulmonary disease. Arch Bronconeumol.

[25] Ghasemi A, Zahediasl S (2012). Normality tests for statistical analysis: a guide for non-statisticians. Int J Endocrinol Metab.

[26] Hautmann H, Hefele S, Schotten K, Huber RM (2000). Maximal inspiratory mouth pressures (PIMAX) in healthy subjects--what is the lower limit of normal?. Respir Med.

[27] Lista-Paz A, Souto Camba S, Vilaró J, Quintela Del Río A, López García A, González Doniz L (2019). Comparative analysis of maximal respiratory pressures with the reference values of a healthy adult population [Spanish]. Fisioterapia.

[28] Beaumont M, Forget P, Couturaud F, Reychler G (2018). Effects of inspiratory muscle training in COPD patients: a systematic review and meta-analysis. Clin Respir J.

[29] Ferreira GD, Costa AC, Plentz RD, Coronel CC, Sbruzzi G (2016). Respiratory training improved ventilatory function and respiratory muscle strength in patients with multiple sclerosis and lateral amyotrophic sclerosis: systematic review and meta-analysis. Physiotherapy.

[30] Laveneziana P, Albuquerque A, Aliverti A (2019). ERS statement on respiratory muscle testing at rest and during exercise. Eur Respir J.

